# Network Proteins of Human Sortilin1, Its Expression and Targetability Using Lycopene

**DOI:** 10.3390/life14010137

**Published:** 2024-01-18

**Authors:** Arun H. S. Kumar

**Affiliations:** Stemcology, School of Veterinary Medicine, University College Dublin, Belfield, D04 V1W8 Dublin, Ireland; arun.kumar@ucd.ie; Tel.: +353-17166-230; Fax: +353-0-17166-104

**Keywords:** Sortilin1, fibrosis, microcalcification, unstable plaque, lycopene

## Abstract

Background: Sortilin1 (SORT1) is a ubiquitously expressed transporter involved in sorting or clearing proteins and is pathologically linked to tissue fibrosis and calcification. Targeting SORT1 may have potential clinical efficacy in controlling or reversing cardiovascular fibrosis and/or calcification. Hence, this study assessed the protein–protein network of human SORT1 and its targetability using known nutra-/pharmaceuticals. Material and methods: Network proteins of human SORT1 were identified using the String database, and the affinity of the protein–protein interaction of this network was analysed using Chimera software (Chimera-1.17.3-mac64). The tissue-specific expression profile of SORT1 was evaluated and assessed for enrichment in different cell types, including immune cells. A library of in-house small molecules and currently used therapeutics for cardiovascular diseases were screened using AutoDock Vina to assess the targetability of human SORT1. The concentration affinity (CA) ratio of the small molecules was estimated to assess the clinical feasibility of targeting SORT1. Results: IGF2R, NTRK2, GRN and GGA1 were identified as high-affinity interaction networks of SORT1. Of these high-affinity interactions, IGF2R and GRN can be considered relevant networks in regulating tissue fibrosis or the microcalcification process due to their influence on T-cell activation, inflammation, wound repair, and the tissue remodelling process. The tissue cell-type enrichment indicated major expression of SORT1 in adipocytes, specialised epithelial cells, monocytes, cardiomyocytes, and thyroid glandular cells. The binding pocket analysis of human SORT1 showed twelve potential drug interaction sites with varying binding scores (0.86 to 5.83) and probability of interaction (0.004 to 0.304). Five of the drug interaction sites were observed to be targetable at the therapeutically feasible concentration of the small molecules evaluated. Empagliflozin, sitagliptin and lycopene showed a superior affinity and CA ratio compared to established inhibitors of SORT1. Conclusion: IGF2R and GRN are relevant networks of SORT1, regulating tissue fibrosis or the microcalcification process. SORT1 can be targeted using currently approved small-molecule therapeutics (empagliflozin and sitagliptin) or widely used nutraceuticals (lycopene), which should be evaluated in a randomised clinical trial to assess their efficacy in reducing the cardiac/vascular microcalcification process.

## 1. Introduction

Sortilin1 (SORT1) is a ubiquitously expressed transmembrane glycoprotein that functions as a sorting receptor in the Golgi apparatus and a clearance receptor on the cell membrane, and it is vital to the functional regulation of cell homeostasis [1,2,3,4]. SORT1 is predominantly found in the Golgi apparatus and endosomes, where it plays a crucial role in various biochemical processes. As a multifunctional receptor, SORT1 is involved in the trafficking and sorting of proteins within the cell, influencing their ultimate destination and subsequent cellular functions. In its role as a sorting receptor, it transports proteins from the Golgi apparatus to their intended target site using the endosomes/lysosomes as cargo, based on pH-based dissociation kinetics [3,4]. Notably, SORT1 is implicated in the regulation of neurotrophic factors such as brain-derived neurotrophic factor (BDNF), impacting neuronal survival and function. Additionally, SORT1 is associated with lipid metabolism, participating in the intracellular transport of lipoproteins. Among the various transporter functions of SORT1, it can scavenge extracellular lipoprotein lipase [5] and promote the mineralisation of extracellular matrix [6], contributing to tissue fibrosis, calcification, and foam cell formation [7,8]. SORT1 seems to favour the intracellular accumulation of lipids and triglycerides through the suppression and/or degradation of adipogenesis factors and lipid efflux transporters [3,7]. The expression of SORT1 is highly enriched in adipocytes [9,10] and smooth muscle cells [7,11], which, together with its contribution to tissue fibrosis and calcification, makes it a vital target in the development of unstable atherosclerotic plaques (UAPs) and vascular calcification. In addition, the expression of SORT1 is reported to be altered in various types of cancer, and its dysregulation is often associated with tumour progression and metastasis. In some instances, SORT1 acts as a mediator of cellular processes that promote cancer growth, such as regulating cell survival pathways and influencing the invasive potential of cancer cells. Additionally, SORT1 also modulates the response to therapeutic interventions, impacting the efficacy of cancer treatments. The intricate involvement of SORT1 in signalling pathways related to tumour development suggests its potential as a biomarker for cancer prognosis and as a target for novel therapeutic strategies.

An increased expression of SORT1 is observed in calcified arteries of humans [7,8]. Consistent with this, SORT1-knockout mice show reduced arterial calcification without impacting bone mineral density [7,8]. This suggests a specific role of SORT1 in the pathological microcalcification process, without disturbing physiological bone mineralisation. SORT1 is reported to transport alkaline phosphatase (ALP) to the extracellular vesicles (EVs) [12] and trigger the mineralisation of the extracellular matrix (specifically collagen), which leads to a functional compromise of an otherwise elastic tissue [6,7]. The role of SORT1 in microcalcification can collaterally contribute to the formation of UAPs through its synergistic effects on the secretion of proinflammatory cytokines (IL6 and IFN gamma) from macrophages [13,14]. Inflammation and microcalcification are hallmarks of UAPs to which SORT1 seems to have a direct contributory role, independent of its effects on lipoprotein metabolism [7,13]. Interestingly the contribution of SORT1 to UAPs is also independent of plasma cholesterol levels [7,13]. The microcalcification promotion role of SORT1 is not limited to vascular smooth muscles, as recently, SORT1 was reported to promote aortic valve fibrosis and calcification following mechanical injury [8]. It appears that following tissue injury, the transporter function of SORT1 facilitates extracellular matrix deposition (EV-dependent and -independent mechanisms), which is further reinforced by ALP-induced calcification to minimise the impact of the tissue injury. During this calcification process, if the endogenous pool of progenitor cells is not efficient enough to replace the damaged tissue, then the calcified tissue will persist through further mineralisation. Hence, there seems to be a transient window during which the activity of SORT1 can be pharmacologically interfered with to promote tissue regeneration (rather than calcification) by either endogenous or exogenous progenitor cells. Its diverse functions highlight the significance of SORT1 in maintaining cellular homeostasis and underscore its potential implications in various physiological and pathological conditions, including neurodegenerative disorders, tumours, and metabolic diseases. Understanding the biochemical intricacies of SORT1 contributes to advancing our knowledge of cellular processes and holds promise for developing targeted therapies for associated conditions. Hence, to address the pharmacological interference of SORT1, this study assessed its protein network, expression profile in human tissues, and targetability using small molecules.

## 2. Materials and Methods

The network protein analysis of human SORT1 was conducted as reported before using the STRING database (https://string-db.org (accessed on 6 September 2023) to observe its functional protein–protein interactions [15,16]. In this study, only the top 10 networks of SORT1 identified in the STRING database were used for further analysis. The protein networks were identified and reviewed in the UniProt database, and the most resolved protein structure based on the full length of protein and atomic resolution was selected for analysis in this study. The PDB code or AlphaFold structure of the selected protein was used to import the protein structure onto the Chimera software (Chimera-1.17.3-mac64), and the number of hydrogen bonds (H-bond) formed between SORT1 and its associated network proteins at a 10 Armstrong (10A) distance was evaluated. A heatmap of the number of H-bonds formed between SORT1 and its associated network proteins was generated to identify the high-affinity interactions.

The protein expression profile of SORT1 across diverse human tissues was systematically retrieved through text mining from the Human Protein Atlas database (https://www.proteinatlas.org (accessed on: 6 September 2023)). This comprehensive dataset provides valuable insights into the spatial distribution and abundance of the SORT1 protein in various organs and tissues, offering a holistic view of its expression pattern in the human body. In parallel, information on the enrichment of SORT1 in different tissue cell types was also extracted from the Human Protein Atlas database. This additional layer of analysis allows for a more detailed understanding of SORT1’s cellular localisation within specific tissues, shedding light on the potential functional roles it may play in distinct cell types. This nuanced approach contributes to a more comprehensive characterisation of SORT1’s involvement in various physiological processes. An analysis of SORT1 mRNA expression patterns in human immune cells from the same database was also performed as this adds another dimension to our understanding of SORT1’s role in the immune system. Assessing its expression in immune cells provides insights into the potential involvement of SORT1 in immune response modulation, particularly in the context of inflammatory processes and immune cell function.

The binding sites of human SORT1 were identified using the PrankWeb: Ligand Binding Site Prediction tool (https://prankweb.cz/ (accessed on 15 September 2023)). To assess the targetability of SORT1 by small molecules, in this study, the molecular docking of selected drugs approved for the therapeutics of cardiovascular disease were evaluated against the AlphaFold structure of human SORT1 using AutoDock Vina 1.2.0. Briefly, the AlphaFold structure of SORT1 was optimised for molecular docking using AutoDock MGL tools. The isomeric SMILES sequence of the selected small molecules was obtained from the PubChem database and converted into the PDB format using the Chimera software (Chimera-1.17.3-mac64) following the optimisation of the structure for molecular docking [17,18]. The PDB structures of small molecules were further processed using the AutoDock MGL tools for molecular docking, as reported previously [15,16,17,18,19]. The SORT1 AlphaFold structure was also screened against our in-house compound library to identify a high-affinity inhibitor. Moreover, AF38469 and SB203580, which are reported to be orally bioavailable SORT1 inhibitors, were used as reference compounds [3,7,8,9]. To assess the targetability potential of SORT1 by the small molecules, the concentration affinity ratio (CA ratio) for each of the small molecules was calculated as described before [17,19].

## 3. Results

**Figure 1:**
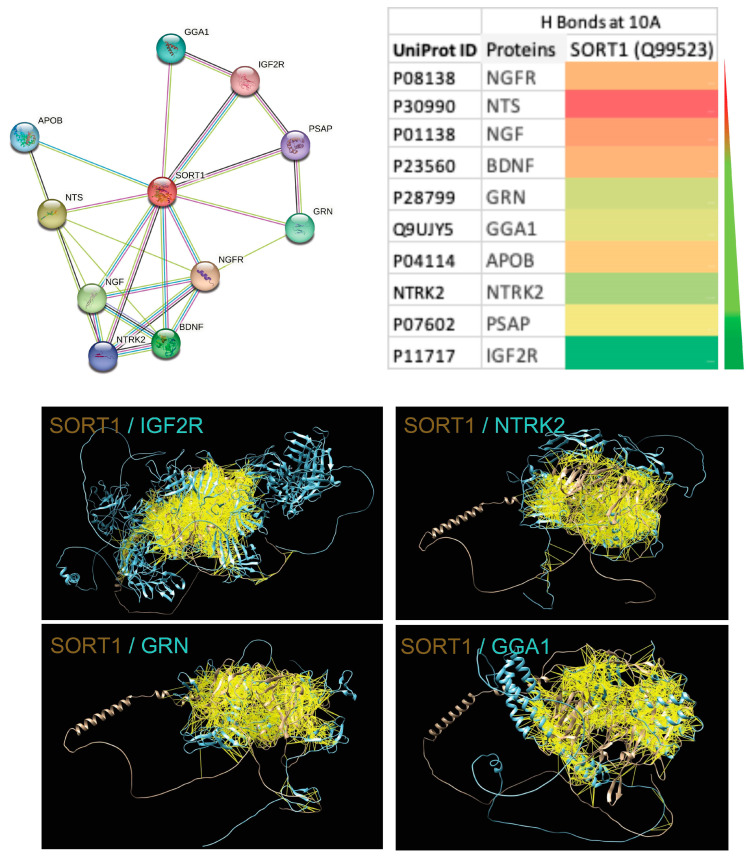
Affinity of human Sortilin1 (SORT1; UniProt ID Q99523) to its network proteins. Network proteins of SORT1 from the string database are shown. The network proteins are represented using their gene code and UniProt ID. Heatmap showing the degree of interaction (number of hydrogen bonds (H Bonds)) between each of the network proteins and SORT1 at a 10 Armstrong (10A) bond distance. Scale: green to red = high to low. The high-affinity network proteins (IGF2R, NTRK2, GRN, and GGA1) in cyan are interacting (yellow lines represent the H Bonds) with human SORT1 (brown).

The network protein analysis of SORT1 in humans showed high-affinity interactions with IGF2R, NTRK2, GRN and GGA1 (Figure 1). IGF2R is a cation-independent mannose-6-phosphate receptor, which facilitates the transport of phosphorylated lysosomal enzymes from the Golgi complex/cell surface to lysosomes and also positively regulates T-cell coactivation [20,21]. As a multifunctional receptor, IGF2R is involved in the regulation of cell growth, differentiation, and survival by modulating the bioavailability of insulin-like growth factors (IGFs). The dysregulation of IGF2R has been associated with several diseases, including cancer, metabolic disorders, and neurodegenerative diseases. NTRK2 is a neurotrophic factor receptor tyrosine kinase that supports the development and maturation of the nervous system, including establishing nerve–nerve or nerve–glial cell synapses. [22,23]. NTRK2, also known as TrkB, is a receptor for brain-derived neurotrophic factor (BDNF) and plays a pivotal role in neuronal survival, development, and function. While NTRK2 is essential for normal neurological processes, its dysregulation has been implicated in neuropsychiatric disorders (depression, anxiety, and schizophrenia), synaptic plasticity, neurotransmitter release, and tumour growth and progression. GRN is a secreted protein that modulates inflammation, wound repair, and tissue remodelling through cytokine-like mechanisms influencing fibroblasts and endothelial cells [24,25]. One of the most notable associations of GRN is with neurodegenerative disorders, particularly frontotemporal dementia (FTD). Mutations in the GRN gene are associated with a familial form of FTD, leading to a reduction in functional GRN levels. This deficiency contributes to the accumulation of abnormal protein aggregates in the brain, leading to neurodegeneration. In cancer, GRN’s involvement is complex, with evidence suggesting both pro-tumorigenic and anti-tumorigenic effects depending on the specific context. GGA1 is a binding protein that facilitates two-way protein sorting and trafficking between the trans-Golgi network (TGN) and endosomes [26]. GGA1 has been associated with neurodegenerative disorders, particularly Alzheimer’s disease. Studies have suggested that GGA1 is involved in the sorting of proteins, including those related to Alzheimer’s pathology, such as the amyloid precursor protein (APP). The dysregulation of GGA1-mediated sorting processes could contribute to the accumulation of amyloid-beta plaques, a hallmark of Alzheimer’s disease. Altered expression levels of GGA1 have been observed in various cancer types, and it appears to be involved in the trafficking of proteins associated with tumour growth and metastasis.

**Figure 2:**
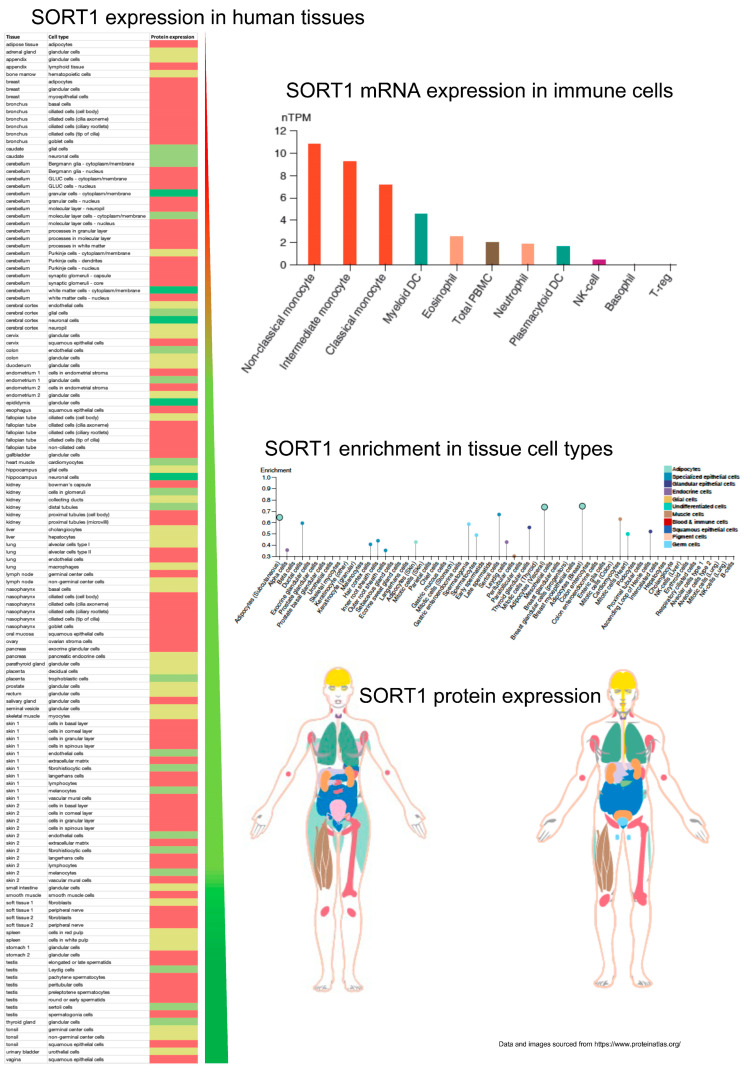
Expression profile of Sortilin1 (SORT1) in human tissues. The quantitative protein expression of SORT1 in various human tissue and cell types is shown as a heat map (Scale: green to red = high to low). The mRNA expression of SORT1 in human immune cells is shown as a bar graph (The expression profile is categorised based on the nTPM ranging from 0 to 12 and colour-coded based on cell category type). The cell-type enrichment (Scale 0 to 1) of the SORT1 protein in human tissues is shown as a vertical line graph and colour-coded based on cell category type. The gender-specific protein expression of SORT1 in various human tissues is shown qualitatively using images.

The analysis of SORT1 protein expression in various human tissues reveals a diverse distribution pattern, with its presence in key organs and tissues. The highest levels of SORT1 expression are observed in the brain, kidneys, skin, colon, placenta, endocrine glands, and testis (epididymis) (Figure 2), suggesting the potential role of SORT1 in the functions of these organs. Moreover, tissue cell-type enrichment analysis provides additional insights into SORT1 expression in specific cell types. Major expression was observed in adipocytes (Figure 2), highlighting its potential involvement in adipose tissue function. Specialised epithelial cells also exhibit significant SORT1 expression (Figure 2), suggesting a role in epithelial tissue homeostasis. Additionally, a high expression in cardiomyocytes indicates a potential impact on cardiac function, while the presence in thyroid glandular cells suggests a role in thyroid physiology. Furthermore, the examination of SORT1 mRNA expression in immune cells points to a significant association with monocyte subsets, particularly non-classical monocytes (Figure 2). This observation underscores the potential role of SORT1 in the immune system, particularly in monocyte-related immune functions. The higher expression in non-classical monocytes implies a specific involvement in immune surveillance and response and the regulation of tumours. Overall, these comprehensive findings shed light on the diverse tissue distribution and cell-type enrichment of SORT1 expression in the human body. Understanding SORT1’s localisation in various tissues and cell types provides a foundation for further investigations into its specific functions and potential implications in physiological and pathological processes.

**Figure 3:**
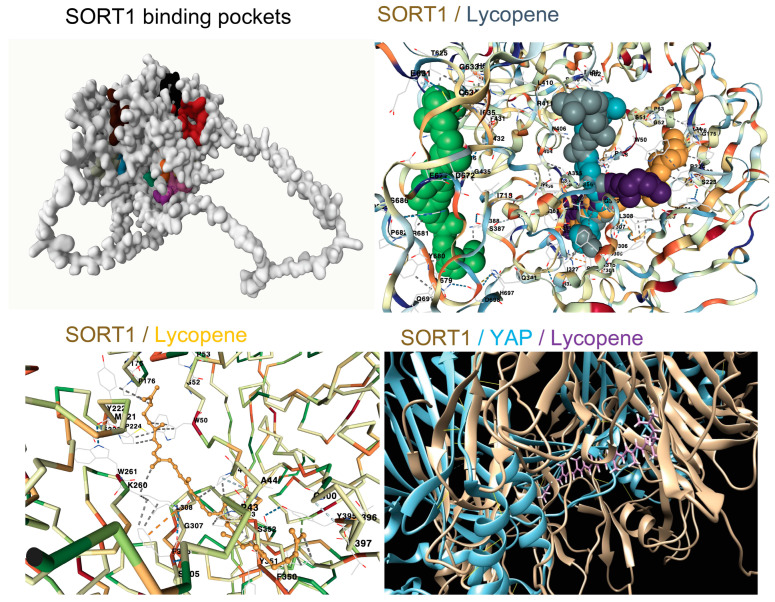
Targetability of human Sortilin1 (SORT1). The twelve binding pockets of SORT1 identified are colour-coded and represented in 2D (the relative area of the colour indicates the binding score, which ranged from 0.86 to 5.83). The molecular docking of lycopene (ball and stick model) with human SORT1 with its five interaction sites is shown. The bottom left panel shows the highest affinity interaction between lycopene (stick model) with human SORT1. The bottom right panel shows the interaction sites of lycopene (purple stick model) between human SORT1 (brown colour) and yes-associated protein 1 (YAP; cyan colour).

The binding pocket analysis of human SORT1 has identified twelve potential drug interaction sites, each characterised by varying binding scores ranging from 0.86 to 5.83 and probabilities of interaction spanning from 0.004 to 0.304 (Figure 3). These scores and probabilities provide crucial information about the likelihood and strength of ligand binding to SORT1. The top-ranking binding pocket, with a score of 5.83, is of particular interest. It encompasses 11 amino acids (A432, A631, A634, A635, A636, A637, A667, A669, A671, A672, A718) and exhibits 48 solvent-accessible surface (SAS) points. The pocket’s dimensions in three-dimensional space (x, y, and z: −29.6892, 3.1138, and −12.6381) further define its spatial characteristics. This information is crucial for understanding the structural details of the binding site and can guide the design of targeted therapeutic molecules. The second-ranking binding pocket, with a score of 4.6, is also noteworthy. It consists of 17 amino acids (A341, A377, A386, A387, A388, A435, A674, A675, A676, A677, A678, A679, A680, A681, A691, A697, A698) and exhibits 66 SAS points. The three-dimensional coordinates (x, y, and z: −31.2511, −5.5778, and 0.9165) provide spatial insights into this binding site. While not as highly ranked as the top pocket, its structural characteristics suggest potential significance in ligand binding and therapeutic targeting. The remaining binding pockets, with scores below 3 and probabilities of interaction less than 0.092, are considered less favourable for drug interaction and are not further described. Overall, these findings offer valuable information for the development of targeted drugs or therapeutic agents that could modulate SORT1 function, potentially influencing its role in various physiological and pathological processes.

**Figure 4:**
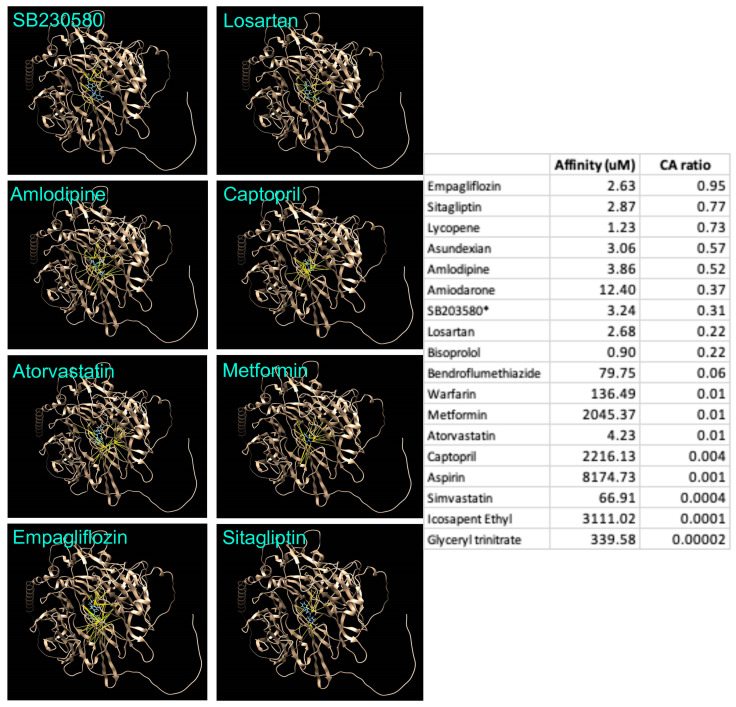
Affinity of small molecules with human Sortilin1 (SORT1). The affinity and concentration–affinity (CA) ratio value of various small molecules against SORT1 is summarised in the table. The images show the degree of interaction (number of hydrogen bonds; yellow lines) and the interaction sites between the selected small molecules and SORT1 (brown colour).

The screening of the in-house ligand library against human SORT1 showed the high affinity of lycopene against five of the SORT1 binding pockets (Table 1, Figure 3). In a recent study, SORT1 was reported to contribute to the calcification of human aortic valves involving MAPK and YAP pathways that drive the trans-differentiation of functional cells to a myofibroblastic-osteogenic phenotype [8]. Lycopene was observed to bind at several SORT1–YAP interaction sites (Figure 3), suggesting its potential to interfere with SORT1–YAP signalling. The potential of various cardiovascular therapeutics to target SORT1 was also analysed (Figure 4). Empagliflozin and sitagliptin showed a therapeutically feasible affinity and CA ratio against SORT1, similar to that of lycopene (Figure 4). Among the small molecules tested, bisoprolol had the highest affinity to SORT1; however, considering its very low CA ratio, the feasibility of achieving therapeutic efficacy without undesired effects (safety) is unlikely. Two of the orally bioavailable SORT1 inhibitors reported in the literature [3,8], i.e., SB203580 (3.24 µM) and AF38469 (638.76 µM), showed an affinity to SORT1 that was inferior to lycopene (1.23 µM) (Figure 4).

## 4. Discussion

The identification of SORT1 as a driver of microcalcification processes in cardio-valvular and cardiovascular diseases, irrespective of lipid metabolism and cholesterol levels, underscores its pivotal role in pathophysiological mechanisms beyond traditional risk factors [1,7,8,27]. In light of this, our study delved into the potential targetability of SORT1 and discovered new therapeutic small molecules, such as lycopene, empagliflozin, and sitagliptin, which exhibit affinity to SORT1 within therapeutically viable ranges. Expanding the clinical implications, these findings are not confined to cardiovascular contexts alone. SORT1’s involvement in diverse physiological processes positions it as a potential therapeutic target for a broader spectrum of diseases, including neurodegenerative conditions and various cancers. This broader perspective aligns with the growing recognition of shared molecular pathways through network proteins of SORT1 and risk factors across seemingly disparate disease categories. This study proposes small molecules targeting SORT1 for clinical trial evaluation, as their potential impact reaches beyond cardiovascular health. The prospect of targeting SORT1 opens avenues for novel therapeutic strategies addressing not only heart-related disorders but also neurodegenerative diseases, where protein misfolding and aggregation are prevalent, as well as various cancers, where dysregulated signalling pathways contribute to tumorigenesis. In essence, this study not only advances our understanding of SORT1’s role in cardiovascular diseases but also introduces promising avenues for developing therapeutics that could have far-reaching implications across diverse medical disciplines. By exploring the potential of SORT1-targeted interventions, we move closer to a holistic approach to addressing complex diseases that share common underlying molecular pathways.

The comprehensive analysis of SORT1’s major network proteins in this study has shed light on collective support for SORT1’s transporter function. Notably, the SORT1–GRN network emerges as a focal point of interest, particularly in the context of the microcalcification process. The interplay between SORT1 and GRN holds special significance due to its intricate involvement in various biological processes. Within the context of microcalcification, the SORT1–GRN network gains prominence by orchestrating a synergistic modulation of inflammation, wound repair, and tissue remodelling processes. GRN, also known as progranulin, plays a pivotal role in mediating inflammation, fostering tissue repair, and influencing tissue remodelling. This synergistic relationship becomes particularly relevant in the context of microcalcification, where inflammation, tissue repair, and remodelling processes are intricately linked to the progression of calcification. The SORT1-facilitated extracellular matrix deposition, coupled with ALP-induced tissue calcification, underscores the intricate molecular mechanisms driving the microcalcification process. SORT1, acting as a transporter, likely contributes to extracellular matrix dynamics, creating a microenvironment conducive to tissue calcification [24,25,28]. Meanwhile, the involvement of GRN adds another layer of complexity, influencing the inflammatory milieu and tissue remodelling processes that contribute to the microcalcification cascade. Expanding the scope of relevance, these intricate molecular interactions within the SORT1–GRN network may also have implications in cancer biology. Dysregulated tissue remodelling, inflammation, and extracellular matrix dynamics are common features in cancer progression, invasion, and metastasis. Therefore, understanding the SORT1–GRN network’s role in microcalcification provides insights that extend beyond cardiovascular diseases to potentially include aspects of cancer pathophysiology. Further exploration of these molecular networks could uncover novel therapeutic targets applicable across diseases with shared underlying mechanisms, contributing to the development of targeted interventions for both cardiovascular and oncological conditions. A recent study has reported the role of MAPK and YAP pathways in SORT1-induced aortic valve calcification following mechanical injury [8]. In the context of cardiovascular health, the involvement of MAPK and YAP pathways in SORT1-induced aortic valve calcification emphasises the complexity of signalling cascades contributing to pathological processes. These pathways are known to regulate cellular responses to various stimuli, including mechanical stress. However, it is possible that the biological response to acutely induced mechanical injury may differ from subacute/chronic injury classically seen with cardio-valvular and cardiovascular diseases in humans. The increased expression of SORT1 observed in calcified arteries of humans [7,29,30] does suggest that some overlap exists in the biological response to acute, subacute and chronic injury to tissues. This concept was validated by a preclinical study simulating acute mechanical tissue injury [8] and pharmacological interventions, demonstrating the efficacy of orally bioavailable SORT1 inhibitors in reducing the incidence of the microcalcification process [7,8,31,32]. The comparison of the affinity of these SORT1 inhibitors with lycopene, empagliflozin and sitagliptin in this study suggests that potential superior efficacy outcomes are possible using the novel small molecules identified in this study, which will be interesting to evaluate in randomised clinical trials. The therapeutic use of empagliflozin [33,34] and sitagliptin [35,36] in patients who are at higher risk of developing tissue calcification should allow for the rapid testing of its clinical efficacy in reducing the incidence of calcified aortic valves and/or unstable plaques. While the primary focus is often on cardiovascular conditions such as calcified aortic valves and unstable plaques, extending these investigations to include neurodegenerative diseases and cancer could reveal broader applications for these drugs. In the context of neurodegenerative diseases, where protein aggregation and tissue remodelling play critical roles, targeting tissue calcification may offer neuroprotective effects. The use of empagliflozin and sitagliptin could potentially modulate underlying inflammatory processes, tissue remodelling, and calcification, thereby influencing the progression of neurodegenerative disorders. Exploring their therapeutic efficacy in this context may open new avenues for interventions aiming at slowing or mitigating the impact of neurodegenerative diseases. Similarly, in oncology, where dysregulated tissue remodelling and calcification contribute to tumour progression, the use of these drugs could have multifaceted benefits. By addressing the shared molecular pathways implicated in both cardiovascular and oncological conditions, empagliflozin and sitagliptin might offer therapeutic strategies that extend beyond their traditional use in diabetes management. The rapid testing of the clinical efficacy of empagliflozin and sitagliptin in reducing the incidence of calcified aortic valves, unstable plaques, and potentially influencing neurodegenerative diseases and cancer could usher in a paradigm shift in the approach to these complex and interconnected health challenges. Such investigations not only present the opportunity to repurpose existing medications for novel therapeutic applications but also highlight the importance of understanding shared pathophysiological processes that span seemingly distinct medical conditions. Further, as lycopene is generally regarded as a safe nutraceutical [37,38], a coformulation of lycopene/empagliflozin or lycopene/sitagliptin can also be evaluated for synergistic therapeutic efficacy. Concerns are also expressed about achieving a therapeutic concentration of lycopene in circulation [39,40]. However, the large volume of distribution of lycopene (150 to 1400 L or 2–19 L/kg), together with its high lipo-solubility, suggests that it is extensively distributed into peripheral tissues [39,40]. Additionally, population studies have shown serum lycopene concentrations of >0.7 µM, suggesting that a SORT1-targetable concentration (CA ratio ~= 1) is achievable. In addition to the SORT1 inhibitors, other allosteric approaches such as small molecules that can interfere with SORT1–YAP or SORT1–GRN signalling may also be developed as therapeutics for the clinical management of calcified valves/arteries. 

The expression of SORT1 in cardiomyocytes is consistent with its pathological role in valvular calcification [8,41], although its lower expression in vascular smooth muscles contradicts its reported role in arterial calcification. In this context, the contributory role of non-classical monocytes (myeloid cells) in the formation of viable vascular smooth muscles can be a mechanism by which SORT1 leads to vascular classification [42,43]. The constant recycling of vascular smooth muscle cells by myeloid cells is an essential process in retaining optimal vascular physiology, and the role of SORT1 in this process is unclear. However, following mechanical injury to arteries, the myeloid cells promote the remodelling of vascular smooth muscles under an inflammatory milieu, and paraphs SORT1 plays an active role in this process [42,43]. The highest expression profile of SORT1 mRNA in the monocyte subpopulation among all immune cells, together with its expression in calcified vascular tissue, supports its role in paraphs SORT1 pathological vascular remodelling process, eventually leading to microcalcification. Non-classical monocytes are known for their involvement in tissue repair and inflammation and might play a key role in the vascular calcification process mediated by SORT1. It is plausible that SORT1, through its interaction with non-classical monocytes, influences their differentiation into vascular smooth muscle cells, contributing to the pathogenesis of arterial calcification. This intricate interplay between SORT1, non-classical monocytes, and vascular smooth muscle cells could represent a novel mechanism by which SORT1 influences vascular calcification. The intricate involvement of SORT1 in cellular processes extends beyond the cardiovascular system. In neurodegenerative diseases, where aberrant protein aggregation and inflammatory responses are prevalent, SORT1’s influence on monocytes and tissue remodelling may contribute to disease progression. Similarly, in cancer, where interactions between the tumour microenvironment and immune cells are crucial, SORT1-mediated effects on monocyte behaviour could impact the tumour’s ability to manipulate its surroundings. 

SORT1 is highly expressed in adipocytes, which is consistent with several studies linking the disturbed lipid metabolism with the valvular and arterial calcification process [3,44,45]. However, some preclinical studies have also reported lipid- and cholesterol-independent mechanisms of SORT1 in inducing tissue calcification [41,46,47,48,49]. The lipid-independent role of SORT1 on tissue calcification is of specific interest in the context of UAPs, as none of the therapeutics currently used for lipid management have been shown to reduce the risk associated with UAP vascular pathology [50,51,52]. Hence, targeting SORT1 with or without lipid management therapeutics can prove to be beneficial in improving vascular physiology by reducing the microcalcification process. The potential to target SORT1 using the currently approved small-molecule therapeutics (empagliflozin and sitagliptin) or widely used nutraceuticals (lycopene) identified in this study is particularly promising and should be evaluated in a randomised clinical trial to assess its efficacy in reducing cardiac/vascular microcalcification process.

In conclusion, the interplay between IGF2R and GRN networks with SORT1, as regulators of tissue fibrosis or the microcalcification process, introduces a complex but potentially targetable axis with implications across various medical domains. Recognising the significance of SORT1 in these pathways prompts consideration for therapeutic interventions, and the identification of potential agents, such as empagliflozin, sitagliptin, and lycopene, adds a practical dimension to this exploration. In the context of cardiovascular health, particularly the cardiac and vascular microcalcification process, targeting SORT1 with empagliflozin and sitagliptin presents an intriguing proposition. These approved small-molecule therapeutics have established safety profiles, and their potential efficacy in mitigating microcalcification could offer novel therapeutic avenues. Empagliflozin, a sodium-glucose cotransporter-2 (SGLT2) inhibitor, and sitagliptin, a dipeptidyl peptidase-4 (DPP-4) inhibitor, have demonstrated cardiovascular benefits beyond glycaemic control. Evaluating their impact on the microcalcification process in a randomised clinical trial could provide valuable insights into their broader therapeutic potential in cardiovascular diseases. Lycopene, a widely used nutraceutical, adds a nutritional aspect to potential SORT1-targeted interventions. Its inclusion in the therapeutic repertoire stems from its antioxidant and anti-inflammatory properties. While traditionally associated with its role in cancer prevention, lycopene’s potential in modulating tissue fibrosis or microcalcification processes, especially in the cardiovascular system, deserves investigation. Given the intricate involvement of SORT1 in multiple common pathways, evaluating the efficacy of empagliflozin, sitagliptin, and lycopene in mitigating neurodegenerative processes or influencing cancer progression could reveal unexpected therapeutic benefits. The shared molecular pathways and cellular processes implicated in these diverse diseases highlight the potential for multifaceted interventions that transcend traditional therapeutic boundaries. In summary, a randomised clinical trial assessing the efficacy of empagliflozin, sitagliptin, and lycopene in reducing tissue microcalcification, particularly in the cardiovascular system, holds significant promise. This exploration not only addresses cardiovascular health but also opens avenues for understanding the broader impact of these interventions on neurodegenerative diseases and cancer, offering a holistic perspective on SORT1-targeted therapeutics.

## Figures and Tables

**Table 1:** Molecular docking of lycopene with human SORT1.

Binding Pocket ID	Binding Energy (kcal/mol)	Cavity Volume (Å^3^)	Centre (x, y, z)	Docking Size (x, y, z)	Amino Acid Sequence
C2	−8.2	1820	5, 7, 3	41, 41, 41	PRO43 ALA44 PRO46 TRP50 GLY52 PRO53 ILE174 GLY175 PRO176 MET221 TYR222 SER223 PRO224 LYS260 TRP261 SER305 PHE306 GLY307 LEU308 PHE350 TYR351 SER352 ILE353 TYR395 THR396 THR397 GLY400
C4	−8.1	666	3, 2, −6		PRO46 LEU47 PRO48 ARG49 SER51 TYR304 SER305 PHE306 GLY307 LEU308 GLY309 PHE314 SER316 ARG325 ILE327 PHE350 TYR351 SER352 ILE353 LEU354 ALA355 ALA356 MET363 ASN406 THR408 SER480 GLU481 PRO482
C5	−8.1	445	−3, −4, 22		PRO43 ALA44 PRO46 LEU47 ARG49 TRP50 PRO224 GLN225 LYS260 TRP261 GLY262 SER263 GLY307 LEU308 GLY309 GLY310 ARG325 GLN347 GLU348 GLN349 PHE350 TYR351 SER352 ILE353 TYR395 THR397 THR398 GLY400
C1	−7.9	3278	−13, 0, 0		PRO46 LEU47 PRO48 ARG49 TRP50 LYS260 SER305 PHE306 GLY307 LEU308 GLY309 PHE314 ALA315 SER316 ILE327 TYR351 SER352 ILE353 LEU354 ALA355 ALA356 MET363 PHE404 ASN406 THR408 LEU410 ARG411 PRO482
C3	−7.0	1039	−32, 1, −7		GLN341 PHE377 SER387 LYS388 PHE431 ASP432 GLY435 ARG436 TYR513 HIS623 THR625 GLU631 GLY633 CYS634 ILE635 GLU671 ASP672 TYR679 TYR680 ARG681 PRO682 ASP685 SER686 GLN691 HIS697 ASP698 ILE718

## Data Availability

Data are contained within the article.
